# The incubation period of COVID-19: a global meta-analysis of 53 studies and a Chinese observation study of 11 545 patients

**DOI:** 10.1186/s40249-021-00901-9

**Published:** 2021-09-17

**Authors:** Cheng Cheng, DongDong Zhang, Dejian Dang, Juan Geng, Peiyu Zhu, Mingzhu Yuan, Ruonan Liang, Haiyan Yang, Yuefei Jin, Jing Xie, Shuaiyin Chen, Guangcai Duan

**Affiliations:** 1 Department of Epidemiology and Health Statistics, College of Public Health, Zhengzhou University, No. 100 Kexue Avenue, Zhengzhou, 450001 Henan People’s Republic of China; 2 Department of Nutrition and Food Hygiene, College of Public Health, Zhengzhou University, No. 100 Kexue Avenue, Zhengzhou, 450001 Henan People’s Republic of China; 3 Infection Prevention and Control Department, The Fifth Affiliated Hospital of Zhengzhou University, No.3 Kangfuqian Street, Zhengzhou, 450052 Henan People’s Republic of China; 4 Henan Key Laboratory of Molecular Medicine, Zhengzhou University, No. 100 Kexue Avenue, Zhengzhou, 450001 Henan People’s Republic of China; 5 Centre for Biostatistics and Clinical Trials (BaCT), Peter MacCallum Cancer Centre, No. 305 Grattan Street, Melbourne, 3000 Victoria Australia

**Keywords:** COVID-19, Incubation period, Meta-analysis

## Abstract

**Background:**

The incubation period is a crucial index of epidemiology in understanding the spread of the emerging Coronavirus disease 2019 (COVID-19). In this study, we aimed to describe the incubation period of COVID-19 globally and in the mainland of China.

**Methods:**

The searched studies were published from December 1, 2019 to May 26, 2021 in CNKI, Wanfang, PubMed, and Embase databases. A random-effect model was used to pool the mean incubation period. Meta-regression was used to explore the sources of heterogeneity. Meanwhile, we collected 11 545 patients in the mainland of China outside Hubei from January 19, 2020 to September 21, 2020. The incubation period fitted with the Log-normal model by the coarseDataTools package.

**Results:**

A total of 3235 articles were searched, 53 of which were included in the meta-analysis. The pooled mean incubation period of COVID-19 was 6.0 days (95% confidence interval [*CI*] 5.6–6.5) globally, 6.5 days (95% *CI* 6.1–6.9) in the mainland of China, and 4.6 days (95% *CI* 4.1–5.1) outside the mainland of China (*P* = 0.006). The incubation period varied with age (*P* = 0.005). Meanwhile, in 11 545 patients, the mean incubation period was 7.1 days (95% *CI* 7.0–7.2), which was similar to the finding in our meta-analysis.

**Conclusions:**

For COVID-19, the mean incubation period was 6.0 days globally but near 7.0 days in the mainland of China, which will help identify the time of infection and make disease control decisions. Furthermore, attention should also be paid to the region- or age-specific incubation period.

**Graphic Abstract:**

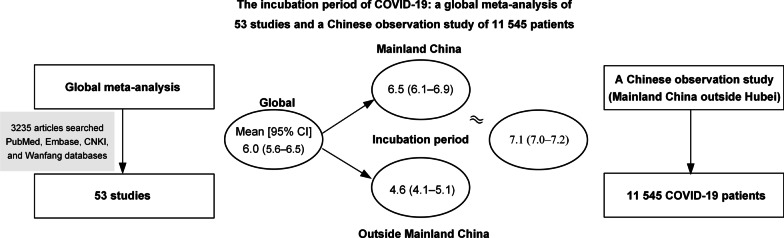

**Supplementary Information:**

The online version contains supplementary material available at 10.1186/s40249-021-00901-9.

## Background

Coronavirus disease 2019 (COVID-19) is caused by severe acute respiratory syndrome coronavirus (SARS-CoV-2). It has spread over 223 countries and has been declared a pandemic on March 11, 2020 by World Health Organization [1]. The number of COVID-19 patients is rapidly increasing globally. More than 113.5 million confirmed cases and 2.5 million deaths were reported globally by March 2, 2021 [1]. The COVID-19 pandemic has become a worldwide public health issue.

The incubation period, known as the interval between initial infection and onset of disease, is an important index to characterize the spread of infectious disease and formulate quarantine measures. For example, the mean incubation period is often used to calculate the reproduction number, and the maximum incubation period is used to determine the duration of quarantine [2]. For COVID-19, its average incubation period has a wide range, ranging from 2.87 days [3] to 17.6 days [4]. Determining the duration of the quarantine is difficult. Several studies have provided mean incubation periods of COVID-19 of about 8 days in the mainland of China outside Hubei Province [5–7]. Some studies have reported shorter mean incubation periods of COVID-19 of about 5 days [8–10]. The incubation period of COVID-19 in previously published studies is inconsistent. In addition, few studies have focused on differences in the incubation period of COVID-19 worldwide. Only one study with 181 COVID-19 patients showed that the patients in the mainland of China had a shorter incubation period than others (4.8 days vs 5.5 days) [11], which seemed to contradict existing evidence. However, although previous meta-analyses have focused on the incubation period of COVID-19, the evidence on the region-specific incubation period is lacking. Therefore, an updated meta-analysis is necessary.

Although existing studies have presented the distribution of the incubation period of COVID-19 in China, they differ from one another probably because of the study population and estimation methods [12]. The first evidence on the incubation period of COVID-19 provides that the mean incubation period of COVID-19 is 5.2 days (95% confidence interval [*CI*] 4.1–7.0) in the early stage of the Wuhan epidemic [13]. The incubation period, defined as the time from the earliest exposure to onset, has a long distribution. Gao et al. revealed a mean incubation period of 9 days using this method [14]. Conversely, short incubation period was reported when it was defined as the time from the latest exposure to onset [8]. The above-mentioned methods inevitably overestimate or underestimate the incubation period of COVID-19. In general, the date of symptom onset is self-reported by the patient, which is considered exact data. Therefore, selecting COVID-19 patients with a single exposure would obtain an accurate incubation period set, which helps understand its distribution.

This study aimed to describe the incubation period of COVID-19. We conducted a meta-analysis to estimate the mean incubation period of COVID-19 globally and collected the information on COVID-19 patients in the mainland of China outside Hubei Province to understand its distribution. We selected patients with the precise date of infection and onset to calculate the accurate incubation period.

## Patients and methods

### Systematic review and meta-analysis

#### Literature search

The meta-analysis was conducted on the basis of the Meta-analysis of Observational Studies in Epidemiology (MOOSE) group [15].

Two authors conducted a literature review on the incubation period of COVID-19 in CNKI, Wanfang, PubMed, and Embase databases between December 1, 2019 and May 26, 2021. The search terms combined incubation period (e.g., “incubation”) and COVID-19 (e.g., “COVID-19,” “SARS-CoV-2,” “2019-nCoV,” “NCP,” “Coronavirus disease 2019,” “severe acute respiratory syndrome coronavirus 2,” “novel coronavirus 2019,” or “novel coronavirus pneumonia,” Additional file 1: Table S1). The language was unrestricted. A total of 3 235 articles were retrieved using the MeSH term and keywords.

#### Selection criteria

**Fig. 1:**
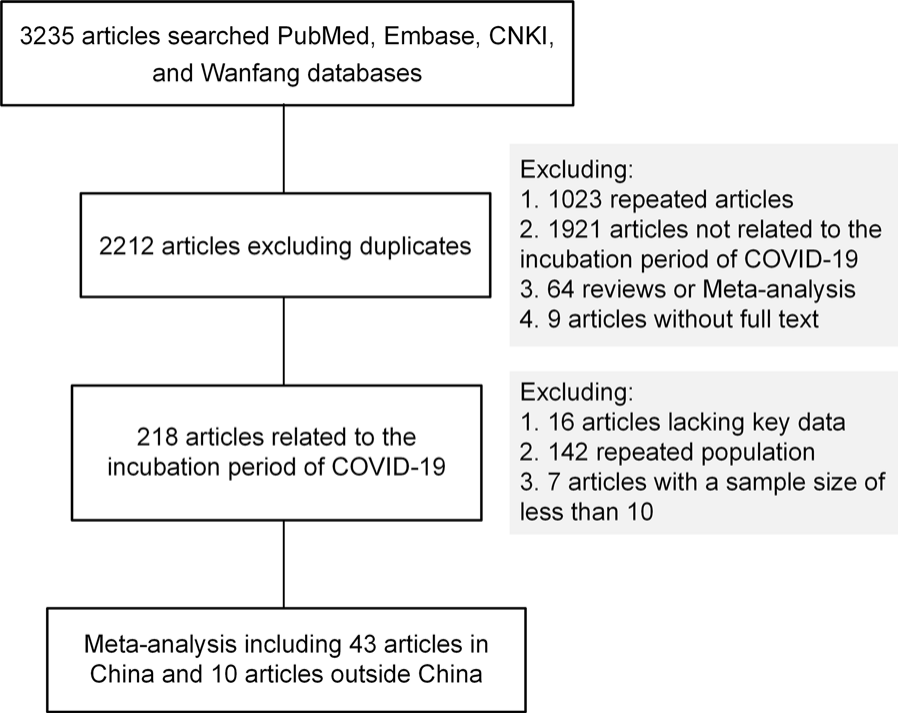
The flowchart of meta-analysis

The irrelevant articles, reviews, and meta-analyses were excluded by title and abstract. Next, the remaining articles were read in detail. Eligible studies should include (1) the mean or median incubation period of COVID-19; and (2) standard deviation (*SD*), interquartile range (IQR), or range. Studies with a sample size of less than ten were excluded. If several eligible studies were identified from the same population, then we will select the article with the largest sample size. When *SD* was not provided, the IQR and range were used to estimate the *SD* [16, 17]. Finally, 53 eligible studies were included in the meta-analysis. Figure 1 presents the flowchart.

#### Data extraction

Information on the first author, country, age, gender, sample of the study, and definition of incubation period was collected. All regions were divided into two groups (the mainland of China and outside the mainland of China). The mean or median age of studies was divided into two groups (< 40 years vs ≥ 40 years). Several estimation methods of incubation period in these eligible studies were identified, which were divided into three groups: (1) method 1 used the interval from the earliest date of exposure to the date of onset of symptoms to estimate incubation period; (2) method 2 used the interval between the exposure interval and the date of onset of symptoms to estimate the incubation period; (3) other methods.

Quality assessment tools to evaluate studies reporting the incubation period of infectious diseases were not available. Thus, the quality assessment of studies was waived.

### Estimations from confirmed COVID-19 patients in the mainland of China outside Hubei Province

#### Study design and participants

This study was approved by the Zhengzhou University Medical Ethics Committee (Zhengzhou, China). The information on all cases was collected from publicly available sources, and informed consent was waived.

By September 21, 2020, a total of 17 168 patients with COVID-19 have been diagnosed with positive nucleic acid and clinical symptoms in the mainland of China outside Hubei Province. Among them, the available information of 11 545 patients can be collected from the national and local health commissions in China [18]. We extracted patients’ data, including age, sex, exposure history, dates of the nucleic acid positive, dates of onset systems, dates of the first treatment, dates of diagnosis, and official reports, to describe the characteristics of cases. All 11 545 patients were included to estimate the incubation period.

We also screened 218 patients with the precise date of infection and onset from 11 545 patients according to the following criteria to accurately calculate the incubation period in subgroup analysis: (1) a clear exposure history, such as contacting with a confirmed patient, traveling to Wuhan, or visiting a place with confirmed cases; (2) have a single short exposure (the earliest date of exposure = the date of latest exposure); (3) have a clear date of exposure; and (4) have a clear date of onset systems (such as fever, cough, and fatigue). However, patients with only imaging results or positive nucleic acid were excluded. A total of 4242 patients had no clear date of onset; 11 045 patients had no single short exposure or a clear date of exposure; and 74 patients had only symptoms of imaging result. Finally, a total of 218 patients met the screening criteria.

#### Definition of case and variables

A patient with positive nucleic acid and clinical symptoms was diagnosed as a COVID-19 case [19]. High-throughput sequencing or real-time RT-PCR assay was used to detect nucleic acid in nasopharyngeal swab specimens.

The incubation period was defined as the time interval between the date of infection and the onset of disease. Among the 11 045 patients, the onset of disease was clear in 7 303 patients and unclear in 4242 patients. Previous evidence suggested that the mean time from onset to first treatment was 2.5 days, and the mean time from onset to diagnosis was 5.4 days [18]. Therefore, for the 4242 patients with an unclear date of onset, we defined date of onset as the date of first treatment minus 2.5 days (*n* = 2168) or the date of diagnosis minus 5.4 days (*n* = 2074). The date of infection was between the date of the earliest exposure and the date of the latest exposure. When the date of the earliest exposure was equal to the date of the latest exposure, the date of infection was the date of exposure; otherwise, it was represented as doubly interval-censored data [20]. Right exposure date (the latest exposure) was at least 1 day earlier than the date that nucleic acid positive or onset of disease was reported [12]. When it was not reported, right exposure date was defined as the date of the nucleic acid positive or the date of onset of disease minus 1 day. Considering most incubation periods of COVID-19 were shorter than 14 days, when the date of the earliest exposure was not reported, or the exposure interval was longer than 14 days, we assumed an exposure interval of 14 days.

### Statistical analysis

In the meta-analysis, the mean incubation period and its *SD* undergone a natural logarithmic transformation. Previous evidence suggested that the Log-normal distribution was the best fit for an incubation period of COVID-19 [18, 21, 22]. The log (incubation period) and its standard error were pooled using the DerSimonian and Laird random-effect model. Mean and *SD* on the original scale were calculated from pooled estimates. Heterogeneity was quantified using the *I*^2^ statistic and investigated by conducting subgroup analyses (such as the regions, average age of patients, ratio of male to female, and study sample). Meta-regression was used to compare differences in heterogeneity among subgroups. Begg’s test and Egger’s test were used to identify the publication bias.

We used mean (95% *CI*) for continuous variables and count (percentage) for categorical variables. The distribution curve of the incubation period fitted with a Log-normal model by the “coarseDataTools” package [20]. Wilcoxon signed-rank test was used to compare differences in subgroup analysis by age and gender. A false discovery rate was used to adjust the *P*-value for multiple comparisons [23]. Sensitivity analysis explored the impact of different definitions of date of onset.

Statistical analyses were conducted using R version 4.0.2 (R Foundation for Statistical Computing, Vienna, Austria) and STATA v12.0 (StataCorp LLC, Texas, USA). Two-sided *P* < 0.05 indicated statistical significance.

## Results

### Pooled mean incubation period in meta-analysis

A total of 3235 articles were retrieved using the MeSH term and keywords. The authors identified 218 articles that reported the incubation period of COVID-19. Among them, 197 articles were from China, and four articles involved multiple countries. The other 17 articles were from Argentina [24], Brunei [25], Thailand [26], France [27, 28], Vietnam [29], Germany [30, 31], India [32, 33], Singapore [34, 35], Korea [3, 36, 37], Saudi Arabia [38], and Uganda [39]. There were 88 articles in English and 130 articles in Chinese (Fig. 1).

**Fig. 2:**
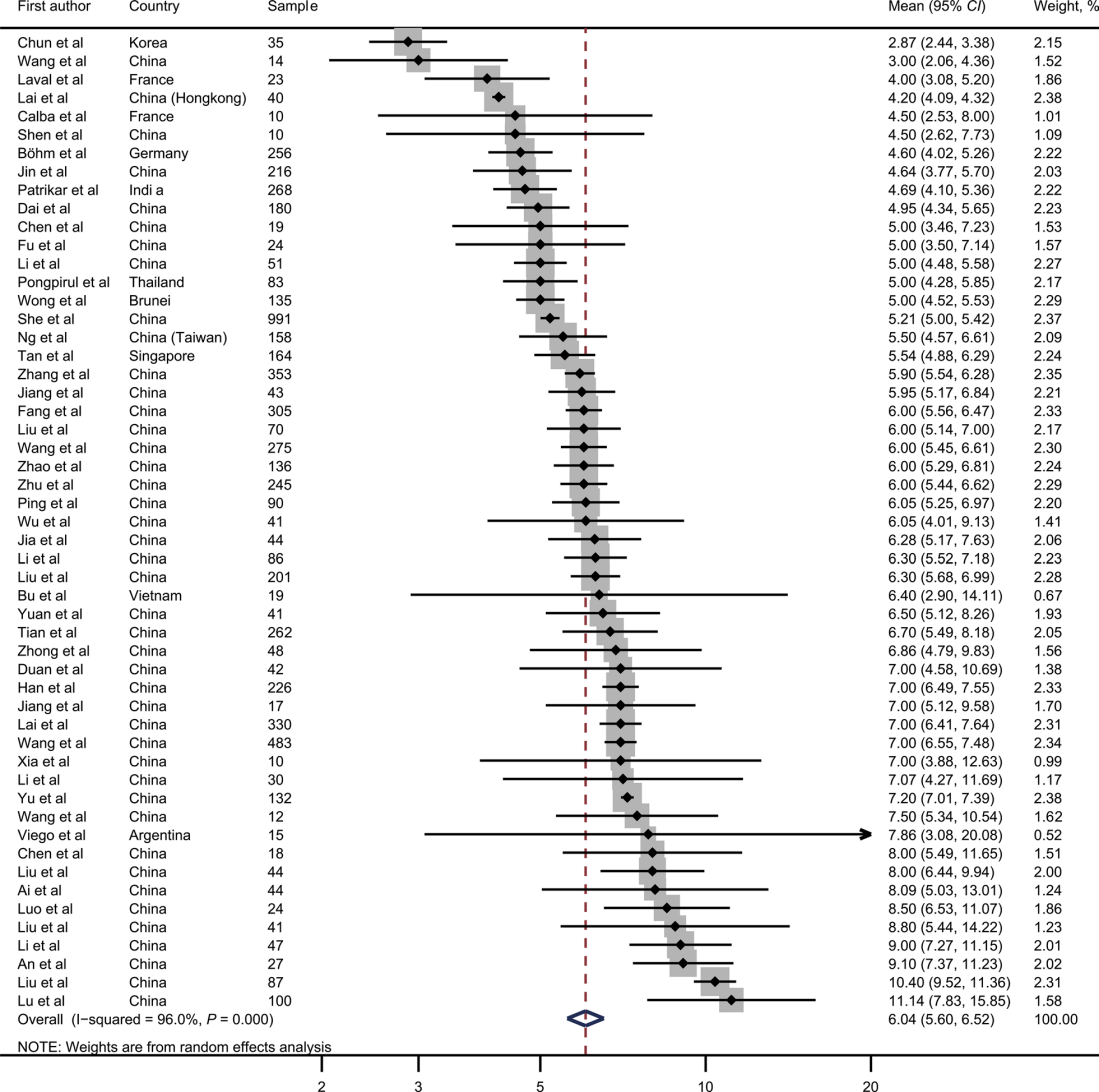
The Forest plot for study-specific mean incubation period of Coronavirus disease 2019. *CI* confidence interval

The meta-analysis included 53 articles, of which 43 were from China [40–83] and ten were outside China [3, 24–30, 32, 34] (Table 1). Figure 2 presents the forest plot of incubation periods. The pooled value was 6.0 days (95% *CI* 5.6–6.5, *I*^2^ = 96.0%, *P* < 0.001) globally. In subgroup analysis, Table 2 and Additional file 2: Figures S1–S8 showed the forest plot for the mean incubation period of COVID-19 by region, age, gender, sample size, estimation method of the date of infection, estimation method of the date of symptom onset, estimation method of the incubation period, and published language. The region, estimation method of the date of infection, and published language were significantly related to the incubation period of COVID-19. The incubation period was 6.5 days (95% *CI* 6.1–6.9, *I*^2^ = 90.7%, *P* < 0.001) in the mainland of China and 4.6 days (95% *CI* 4.1–5.1, *I*^2^ = 83.3%, *P* < 0.001) outside the mainland of China (*P*_meta-regression_ < 0.001). The short incubation period used the exposure interval (mean: 4.7 days; 95% *CI* 3.1–7.0) from the earliest date of exposure (mean: 5.7 days; 95% *CI* 5.0–6.4). A potential source of heterogeneity was not identified. Furthermore, no publication bias was identified in this meta-analysis (Additional file 3: Figure S9; both *P* > 0.05 for Begg’s test or Egger’s test).

**Table 1:** Information on the studies included meta-analysis

Author	Country	Case number	Age, years	Gender	Incubation period, days	References
Ai et al	China	44	–	–	8.09 (*SD*: 4.99)	[40]
An et al	China	27	44	11M:16F	9.1 (R: 3–28)	[41]
Chen et al	China	19	–	–	5 (IQR: 3.3–10)	[42]
Chen et al	China	18	44.5	10M:8F	8 (IQR: 4–12)	[43]
Dai et al	China	180	NA	NA	4.95 (*SD*: 2.46)	[44]
Duan et al	China	42	51.48	15M:27F	7 (IQR: 2–13.5)	[45]
Fang et al	China	305	57	146M:159F	6 (R: 1–15)	[46]
Fu et al	China	24	38.92	14M:10F	5 (R: 0–35)	[47]
Han et al	China	226	44.4	97M:129F	7 (IQR: 5–11)	[48]
Jia et al	China	44	46	15M: 29F	6.28 (R: 1–14)	[49]
Jiang et al	China	43	50	15M:28F	5.95 (R: 2–13)	[50]
Jiang et al	China	17	–	6M:11F	7 (R: 1–14)	[51]
Jin et al. 1	China	21^a^	NA	NA	4 (IQR: 3–7)	[52]
		195^b^	NA	NA	5 (IQR: 3–8)	
Lai et al	China	330	47	161M:169F	7 (IQR:4–12)	[53]
Lai et al	China	40	NA	NA	4.2 (IQR: 4.0–4.5)	[54]
Li et al	China	47	35	23M:24F	9 (R: 1–20)	[55]
Li et al	China	30	32.57	30F	7.07 (*SD*: 4.08)	[56]
Li et al	China	86	–	–	6.3 (R: 1–12)	[57]
Li et al	China	51	–	–	5 (R: 2–10)	[58]
Liu et al	China	201	45.2	103M:98F	6.3 (R: 1–20)	[59]
Liu et al	China	87	NA	NA	10.4 (R:2–25)	[60]
Liu et al	China	41	42	19M:22F	8.8 (*SD*: 4.8)	[61]
Liu et al	China	44	–	–	8 (R: 1–19)	[62]
Liu et al	China	70	40	1.13	6 (R: 1–14)	[63]
Lu et al	China	100	37.13	52M:48F	11.14 (*SD*: 6.05)	[64]
Luo et al	China	24	35.3	14M:10F	8.5 (R: 1–14)	[65]
Ng et al	China	158	45	84M:74F	5.5 (*SD*: 3.26)	[66]
Ping et al	China	90	–	–	6.05 (IQR: 3.81–9.59)	[67]
She et al	China	991	43.96	1.16	5.2 (*SD*: 1.9)	[68]
Shen et al	China	10	60.5	6M:4F	4.5 (IQR: 2–6.5)	[69]
Tian et al	China	262	47.5	127M: 135F	6.7 (*SD*: 5.2)	[70]
Wang et al	China	483	NA	NA	7 (IQR: 4–11)	[71]
Wang et al	China	275	49	128M: 147F	6 (IQR: 3–9)	[72]
Wang et al	China	14	NA	NA	3 (IQR:2–5.25)	[73]
Wang et al	China	12	–	–	7.5 (IQR: 6–13.5)	[74]
Wu et al	China	41	38	19M:22F	6.05 (*SD*: 3.84)	[75]
Xia et al	China	10	56.5	6M: 4F	7 (*SD*: 2.59)	[76]
Yu et al	China	132	NA	172M: 161F	7.2 (IQR: 6.4–7.9)	[77]
Yuan et al	China	41	41	25M:16F	6.5 (IQR: 4.0–11.5)	[78]
Zhao et al	China	136	49	NA	6 (IQR: 4–11)	[79]
Zhang et al	China	353	–	–	5.9 (IQR: 3.8–8.6)	[80]
Zhong et al	China	48	41	31M: 17F	6.86 (*SD*: 3.57)	[81]
Zhu et al	China	245	–	–	6 (R: 1–23)	[82]
Chun et al	Korea	35	NA	NA	2.87 (*SD*: 1.64)	[3]
Viego et al	Argentina	15	NA	NA	7.86 (*SD*: 6.38)	[24]
Wong et al	Brunei	135	36	82/53F	5 (R: 1–11)	[25]
Pongpirul et al	Thailand	83	NA	NA	5 (IQR: 3–8)	[26]
Calba et al	France	10	NA	NA	4.5 (IQR: 2–7)	[27]
Laval et al	France	23	–	–	4 (R: 1–13)	[28]
Bui et al	Vietnam	19	NA	NA	6.4 (*SD*: 5.8)	[29]
Böhm et al	Germany	256	NA	NA	4.6 (*SD*: 3.0)	[30]
Patrikar et al	India	268	36.45	162M:106F	6.93 (*SD*: 5.87)	[32]
Tan et al	Singapore	164	44.2	77M:87F	5 (*SD*: 2.3)	[34]

R, range; IQR, interquartile range; *SD*, standard deviation; M, male; F, female; NA, Not available

^a^With gastrointestinal symptom

^b^Without gastrointestinal symptom

**Table 2:** The study-specific incubation period of Coronavirus disease 2019 in the meta-analysis

Subgroup	Number of articles	Number of patients	Mean incubation period (*95% CI*)	*I*^2^ (%)	*P*_her_	*P*_reg_
Region						< 0.001
The mainland of China	41	5459	6.5 (6.1–6.9)	90.7	< 0.001	
Outside the mainland of China	12	1206	4.6 (4.1–5.1)	83.3	< 0.001	
Median or mean age (years)						0.807
< 40	8	669	6.3 (5.9–6.8)	80.3	< 0.001	
≥ 40	21	3442	6.6 (5.3–8.3)	87.5	< 0.001	
Ratio of male to female						0.572
≤ 1	15	1999	6.5 (6.0–7.1)	78.6	< 0.001	
> 1	15	2125	6.3 (5.6–7.2)	93.7	< 0.001	
Sample of study						0.691
< 100	33	1249	6.1 (5.3–7.1)	95.0	< 0.001	
≥ 100	20	5416	5.9 (5.5–6.4)	93.9	< 0.001	
Estimation method of the date of infection						0.045
The earliest of exposure	19	3130	5.7 (5.0–6.4)	96.0	< 0.001	
Exposure interval	4	603	4.7 (3.1–7.0)	98.3	< 0.001	
Others	30	2932	6.6 (6.0–7.2)	90.3	< 0.001	
Estimation method of the date of onset symptoms						0.064
The date of onset symptoms	23	3411	5.6 (5.0–6.3)	97.7	< 0.001	
Other	30	3254	6.4 (5.8–7.1)	91.0	< 0.001	
Estimation method of incubation period						0.090
From the earliest exposure to onset	16	2296	5.7 (5.0–6.5)	95.7	< 0.001	
From the exposure interval to onset	4	603	4.7 (3.1–7.0)	98.3	< 0.001	
Others	33	3766	6.4 (5.9–7.0)	90.5	< 0.001	
Language						0.025
English	31	4354	5.7 (5.1–6.3)	97.5	< 0.001	
Chinese	22	2311	6.6 (6.1–7.3)	81.4	< 0.001	

*P*_heter_ for heterogeneity within each subgroup estimated by the Cochrane Q test

*P*_reg_ for heterogeneity between subgroups using meta-regression analyses

*CI*, Confidence interval

### Estimating the incubation period in the observation study

**Fig. 3:**
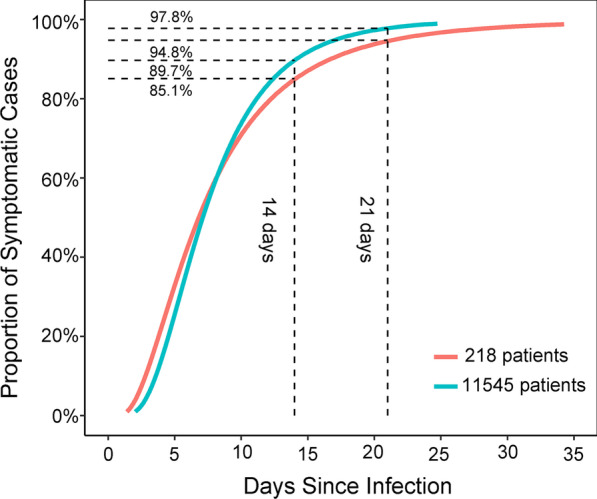
The fitted distribution of incubation period of Coronavirus disease 2019

Table 3 presents that the median age was 45 years (IQR: 33–56), and 5814 males (52.7%) were included. The mean incubation period was 7.1 days (95% *CI* 7.0–7.2) in the total population. Figure 3 shows that 5.4% of patients had an incubation period of less than 3 days; 10.2% of patients had an incubation period of more than 14 days, whereas 2.1% of patients had an incubation period of more than 21 days.

**Table 3:** The incubation period of Coronavirus disease 2019 in the 11 545 patients and in the 218 patients

	11 545 patients		*P*	218 patients		*P*
	N (%)	Mean incubation period (*95% CI*)		N (%)	Mean incubation period (*95% CI*)	
Total	11 545 (100%)	7.1 (7.0–7.2)		218 (100%)	6.8 (6.2–7.4)	
Gender			0.603			0.145
Male	5814 (52.7%)	7.2 (7.0–7.4)		121 (56.3%)	6.3 (5.4–7.2)	
Female	5215 (47.3%)	7.1 (6.9–7.3)		94 (43.7%)	7.5 (6.7–8.4)	
Age, years			< 0.001			0.009
< 18	460 (4.4%)	8.6 (8.0–9.3)		6 (2.8%)	9.3 (5.3–16.3)	
18–40	3789 (36.3%)	7.2 (7.0–7.4)		73 (34.1%)	7.7 (6.6–9.0)	
41–60	4425 (42.3%)	7.1 (6.9–7.2)		98 (45.8%)	5.7 (4.9–6.6)	
> 60	1777 (17.0%)	7.0 (6.6–7.3)		37 (17.3%)	8.0 (6.5–9.8)	
The date of onset			0.319			
Reported date of onset^a^	7303 (63.2%)	7.1 (7.0–7.3)				
Date of first treatment minus 2.5 days^b^	2168 (18.8%)	7.0 (6.7–7.3)				
Date of diagnosis minus 5.4 days^c^	2074 (18.0%)	7.3 (7.0–7.6)				

N, number; CI, Confidence interval

^a^The incubation period was estimated in 7 303 patients with the clear date of onset

^b^The incubation period was estimated in 2 168 patients using the date of first treatment to fill in the missing date of onset

^c^The incubation period was estimated in 2 074 patients using the date of diagnosis to fill in the missing date of onset

As shown in Table 3 and Additional file 4: Figure S10, the mean incubation period was 7.2 days (95% *CI* 7.0–7.4) in male and 7.1 days (95% *CI* 6.9–7.3) in female (*P* = 0.603). Moreover, the mean incubation period was 8.6 days (95% *CI* 8.0–9.3), 7.2 days (95% *CI* 7.0–7.4), 7.1 days (95% *CI* 6.9–7.2), and 7.0 days (95% *CI* 6.6–7.3) in patients under 18 years, 18–40 years, 41–60 years, and over 60 years, respectively (*P* < 0.001).The incubation period was robust among different estimation methods of the date of onset (*P* = 0.319).

Table 3 presents the characteristics of 218 patients with a median age of 47 years (IQR: 35–56); 56.3% of patients were men. The number of patients aged < 18, 18–40, 41–60, and over 60 years was 6 (2.8%), 73 (34.1%), 98 (45.8%), and 37 (17.3%), respectively. A total of 96 patients (44.0%) were infected from January 19 to January 22. Furthermore, a total of 65.6% of patients were exposed to confirmed cases.

**Fig. 4:**
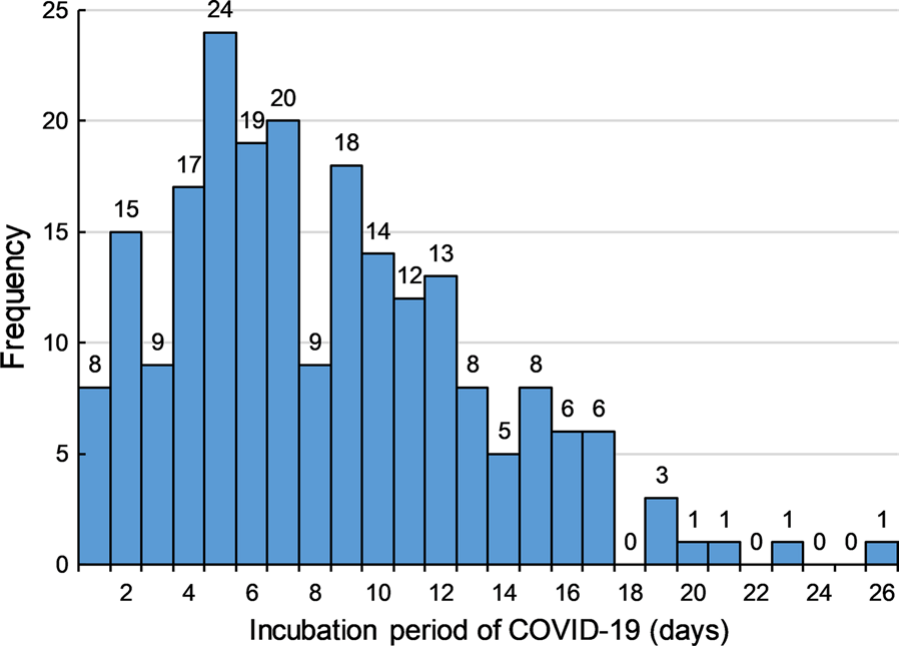
The histogram of individual incubation period of Coronavirus disease 2019 in the 218 patients with precise data

Figure 4 shows the distribution of the accurate incubation period. The median incubation period was 7 days (range: 1–26 days; IQR: 5–11) in 218 patients. Twenty-seven patients had an incubation period of more than 14 days. In the fitted model, the mean incubation period was 6.8 days (95% *CI* 6.2–7.4), and 15% and 5.2% of patients had an incubation period of more than 14 and 21 days, respectively (Fig. 3).

In subgroup analysis (Additional file 5: Figure S11), the incubation period was shorter in patients aged 41–60 years than that in patients aged 18–40 years or over 61 years (41–60 years vs 18–40 years: 6.0 days vs 9.0 days, adjusted *P* = 0.028; 41–60 years vs over 60 years: 6.0 days vs 9.0 days, adjusted *P* = 0.041). The patients exposed to confirmed cases had a longer incubation period than the other groups (9.0 days vs 6.0 days; *P* = 0.015). However, no significant difference in incubation period was observed with regard to sex or date of infection. The fitted curves showed a similar trend to the above-mentioned results.

## Discussion

Several significant findings are obtained in our studies. First, this meta-analysis presented that the mean incubation period of COVID-19 was 6.0 days globally, but it was longer in the mainland of China than in other regions. Next, this study used a large sample to estimate the incubation period of COVID-19. For 11 545 patients, the mean incubation period was 7.1 days; 10.2% and 2.1% of patients had an incubation period of more than 14 and 21 days. Similar results were found in 218 patients set with exact infection and onset data. Finally, only population studies showed that age was related to the incubation period of COVID-19.

The incubation period is a crucial parameter for understanding the epidemiological characteristics of COVID-19. In our study, the mean incubation period of COVID-19 was 6.0 days globally and near 7.0 days in China. The findings of the meta-analysis and observation study were consistent in the Chinese population. Lu et al. reported a mean incubation period of 7.2 days (95% *CI* 6.9–7.5) in 1 158 patients with COVID-19, who also used the interval-censored data [84]. Their result was consistent with the result of this study. The reported mean incubation period varied significantly in previous studies, affecting the estimation method and study population. Some studies had a longer mean incubation period compared with this study, which used the earliest exposure date to estimate the incubation period. For example, Xiao et al. showed a mean incubation period of about 8 days, similar to the findings of Qin et al. [6, 9]. Conversely, other studies that used the last exposure date reported a shorter mean incubation period than this study, ranging from 3 to 5 days [8, 85, 86]. The incubation period was overestimated using the date of the earliest exposure and underestimated using the date of the last exposure date primarily because of the inaccurate date of infection. In addition, studies with shorter maximum incubation periods had shorter mean incubation periods. Tan et al. reported a mean incubation period of 5 days (range: 1–12 days) in 164 patients with COVID-19 [34]. Similarly, a mean incubation period of 5 days (range: 1–11 days) was reported in 135 Brunei patients [25]. On the contrary, a longer mean incubation period was reported in studies with a broader range of incubation period [5, 60]. The distribution type may have a negligible effect on the estimated incubation period of COVID-19, which was relatively robust among different distributions, including Log-normal distribution, Gamma distribution, Weibull distribution, and Erlang distribution [12]. Large samples and accurate data were important for understanding the incubation period. Therefore, our study provided a reliable incubation period of COVID-19, which was robust evidence for understanding SARS-CoV-2 transmission.

Given its potential impact on quarantine strategy, particular attention should be paid to the right tail end of the incubation period [2]. The 14-day quarantine strategy faced a challenge because the incubation period of COVID-19 exceeded 14 days [87]. In this study, among 11 545 patients, more than 10% developed the disease 14 days after infection in the 11 545 patients. Consistent with the results in 218 patients with precise data, the most extended incubation period was 26 days, and 27 patients had an incubation period of more than 14 days. Based on available evidence, the most extended incubation period of COVID-19 was 34 days in Shanghai’s patients, far more than 14 days [4]. The extreme tail end of the incubation period may be affected by the sample size, the knowledge of SARS-CoV-2, and the observation period. Previous studies indicated that 5–10% of patients infected with SARS-CoV-2 had an incubation period longer than 14 days [88]. The above-mentioned evidence indicated that an extended quarantine period was needed to prevent the spread of SARS-CoV-2. When the quarantine interval was longer, more patients will be identified, and the epidemic will be controlled faster. Our findings suggested that the 21-day quarantine strategy will reduce the number of patients without symptoms by 80% compared with the 14-day quarantine strategy.

Take Wuhan as an example, after implementing a strict quarantine strategy of all residents on January 23, the number of new cases with COVID-19 decreased rapidly, with a 50% reduction after 14 days (February 6) and a 75% reduction after 28 days (February 20) [89]. However, the impact of the nucleic acid test on the development of quarantine strategies remained unknown. The nucleic acid test played a vital role in the prevention and control of the COVID-19 pandemic. A previous study reported that 8% of patients had a negative report of nucleic acid test after being quarantined for 14 days [90]. Cai et al. found that over 5% of patients had an incubation period of more than 14 days, which defined the incubation period as the interval from the earliest exposure to laboratory confirmation of COVID-19 or onset of symptoms and signs [91]. We hypothesized that, if only relying on clinical symptoms or nucleic acid test results, then the 14-day quarantine strategy will result in a part of patients not being recognized as confirmed cases. Therefore, adopting a 21-day quarantine strategy was recommended, particularly for places with insufficient detection resources or a high risk of being infected with SARS-CoV-2, such as contacting the patients infected with SARS-CoV-2 or coming from cities or countries where COVID-19 was epidemic.

In addition, the potential patients should be quarantined as soon as possible. We found that about 15% of patients had an incubation period of fewer than 3 days. In India, 25% of patients had an incubation period of fewer than 3 days (25th percentile: 3.0 days) [32]. One-third of patients in Singapore had an incubation period of less than or equal to 3 days [35]. Patients with COVID-19 were infectious before they developed symptoms [92]. Our study found that half of the patients developed symptoms within 7 days after infection. A meta-analysis showed that the mean serial interval of COVID-19 was 5.5 days [93]. Therefore, for COVID-19, the serial interval was shorter than the incubation period. We hypothesized that the spread of SARS-CoV-2 occurred on average 1.5 days before the onset of the disease. If all close contacts are quarantined on the 3rd day after infection, then more than 15% of the people infected with SARS-CoV-2 in close connections may have infected others. When COVID-19 patients and their close and sub-close contacts were quarantined for the first time, further transmission will be terminated.

The incubation period of COVID-19 varied with age. We found that the incubation period was different among age groups. In 218 patients, the incubation period presented a U-shaped curve with increasing age. The middle-aged group (41–60 years) had the shortest incubation period among the other groups, particularly the elderly group (≥ 61 years) and those aged 18–40 years. A similar age-specific distribution of incubation period was reported in the previous study with 136 patients, showing the shortest incubation period in patients aged 45 to 59 years [7]. Another study with 2 555 patients also found a U-shaped curve distribution of incubation period in patients [6]. The mechanism of the effect of age on the COVID-19 incubation period was unclear. Possible explanations include a less intense immune response, a delay in the onset of symptoms, and a shorter exposure time and exposure rate in the elderly and children.

Focusing on the incubation period among different SARS-CoV-2 variants is important. Our finding showed that the incubation period in the mainland of China was longer than that outside the mainland of China (6.5 days vs 4.6 days, *P* < 0.001). Phylogenetic network analysis revealed that the types of SARS-CoV-2 were different among Chinese, Europeans, and Americans in the early stage of the COVID-19 pandemic [94]. Therefore, we hypothesized that the incubation period of COVID-19 may be different among SARS-CoV-2 variants. Recently, an outbreak of SARS-CoV-2 variant B.1.617.2 occurred in Guangzhou, China. In this outbreak, the mean incubation period was 4.4 days (95% *CI* 3.9–5.0), which was shorter than that previously reported in China [95]. These evidences indicated that mutations affected the incubation period of COVID-19. However, more research was needed to explore the relationship of incubation period of COVID-19 with SARS-CoV-2 variants.

However, no difference in incubation period was observed between males and females. The previous study suggested that male was more susceptible to COVID-19, which may be due to the high plasma concentration of ACE2 [96]. However, our study did not observe a difference in incubation period by gender. Nie et al. also showed an insignificant difference in incubation period by gender (5 days vs 4 days; *P* = 0.22) [8]. Yang et al. presented a similar conclusion [97]. The evidence indicated that gender maybe not a factor affecting the incubation period of COVID-19.

Several limitations should be stated in the present study. First, the current meta-analysis did not include studies published in languages other than Chinese or English because of language restrictions. Most of the included studies were from China. Secondly, information bias may also exist. As far as we know, this study had the largest sample size to date, but a part of patients was missing the date of onset. Although no significant difference in incubation periods was observed among the three estimation methods of the date of onset, the estimated date of onset may be biased from the actual date of onset. In addition, most patients had a doubly interval-censored data rather than precise date of infection. For extreme exposure intervals, we limited the exposure interval to 14 days, which might neglect a small part of the extreme incubation period. Third, in the 218 patients with precise data, the proportion of incubation period exceeding 14 days was higher than that in previous studies. This result would obtain an extreme right tail for the incubation period of COVID-19. Finally, the active contact tracing and testing (nucleic acid testing and antibody testing) may truncate the time between exposure to identifying infected persons. Therefore, caution should be taken when formulating quarantine strategies because the estimation of the incubation period did not involve nucleic acid tests and antibody tests.

## Conclusions

This study provides evidence on the incubation period of COVID-19 to understand the transmission of disease and formulate preventive measures. The mean incubation period is 6.0 days globally, but it is longer in the mainland of China (6.5 days) than in other regions (4.6 days). The region- or age-specific incubation period should be paid attention to. Moreover, 10% of patients had an incubation period over 14 days in Chinese population, suggesting that the 14-day quarantine period may not be enough.

## Supplementary Information

**Additional file 1.** Additional Table S1.

**Additional file 2.** Additional Figures S1–S8.

**Additional file 3.** Additional Figure S9.

**Additional file 4.** Additional Figure S10.

**Additional file 5.** Additional Figure S11.

## Data Availability

The data used for this article is available from the corresponding author on reasonable request.

## Abbreviations

ACE2: Angiotensin-converting enzyme 2
*CI*: Confidence interval
COVID-19: Coronavirus disease 2019
CNKI: China National Knowledge Infrastructure
IQR: Interquartile range
2019-nCoV: Novel coronavirus 2019
NCP: Novel coronavirus pneumonia
MOOSE: Meta-analysis of observational studies in epidemiology
R: Range
RT-PCR: Reverse Transcription-Polymerase Chain Reaction
*SD*: Standard deviation
SARS-CoV-2: Severe acute respiratory syndrome coronavirus 2
