# Supplementary material for: The incubation period of COVID-19: a global meta-analysis of 53 studies and a Chinese observation study of 11 545 patients
Source: Infect Dis Poverty. 2021 Sep 17;10:119. doi: 10.1186/s40249-021-00901-9 (PMC8446477; doi:10.1186/s40249-021-00901-9)
Supplement: Supplementary file 1 — Additional file 1. Additional Table S1. [file 40249_2021_901_MOESM1_ESM.docx]

**Table S1**. Systematic literature review search terms and strategy

| **Search terms for PubMed:** #1 AND #2 |
| --- |
| #1 Infectious Disease Incubation Period"[Mesh] OR incubation[Title/Abstract] OR latent[Title/Abstract] |
| #2 COVID-19[Supplementary Concept] OR "severe acute respiratory syndrome coronavirus 2"[Supplementary Concept] OR COVID-19[Title/Abstract] OR "coronavirus disease 2019"[Title/Abstract] OR SARS-CoV-2[Title/Abstract] OR "severe acute respiratory syndrome coronavirus 2"[Title/Abstract] OR 2019-nCoV[Title/Abstract] OR "2019 novel coronavirus "[Title/Abstract] OR "novel coronavirus pneumonia"[Title/Abstract] |
| **Search terms for Embase:** #1 AND #2 |
| #1 incubation time'/exp OR incubation:ab,ti OR 'latent period'/exp OR 'latent':ab,ti |
| #2 'coronavirus disease 2019'/exp OR 'severe acute respiratory syndrome coronavirus 2'/exp OR 'covid-19':ab,ti OR 'coronavirus disease 2019':ab,ti OR 'sars-cov-2':ab,ti OR 'severe acute respiratory syndrome coronavirus 2':ab,ti OR '2019 novel coronavirus ':ab,ti OR '2019-ncov':ab,ti OR 'novel coronavirus pneumonia':ab,ti |
| **Search terms for CKNI:** #1 AND #2 |
| #1 TKA='潜伏期' OR SU='潜伏期' |
| #2 TKA='新型冠状病毒' OR TKA='新冠肺炎' OR TKA='SARS-CoV-2' OR TKA='COVID-19' OR TKA='2019-nCov' |
| **Search terms for Wanfang database:** #1 AND #2 |
| #1 主题:(潜伏期) or 题名或关键词:(潜伏期) |
| #2 题名或关键词:(新型冠状病毒 or 新冠肺炎 or SARS-CoV-2 or COVID-19 or 2019-nCov) |
