# Supplementary material for: The incubation period of COVID-19: a global meta-analysis of 53 studies and a Chinese observation study of 11 545 patients
Source: Infect Dis Poverty. 2021 Sep 17;10:119. doi: 10.1186/s40249-021-00901-9 (PMC8446477; doi:10.1186/s40249-021-00901-9)
Supplement: Supplementary file 2 — Additional file 2. Additional Figures S1–S8. [file 40249_2021_901_MOESM2_ESM.docx]

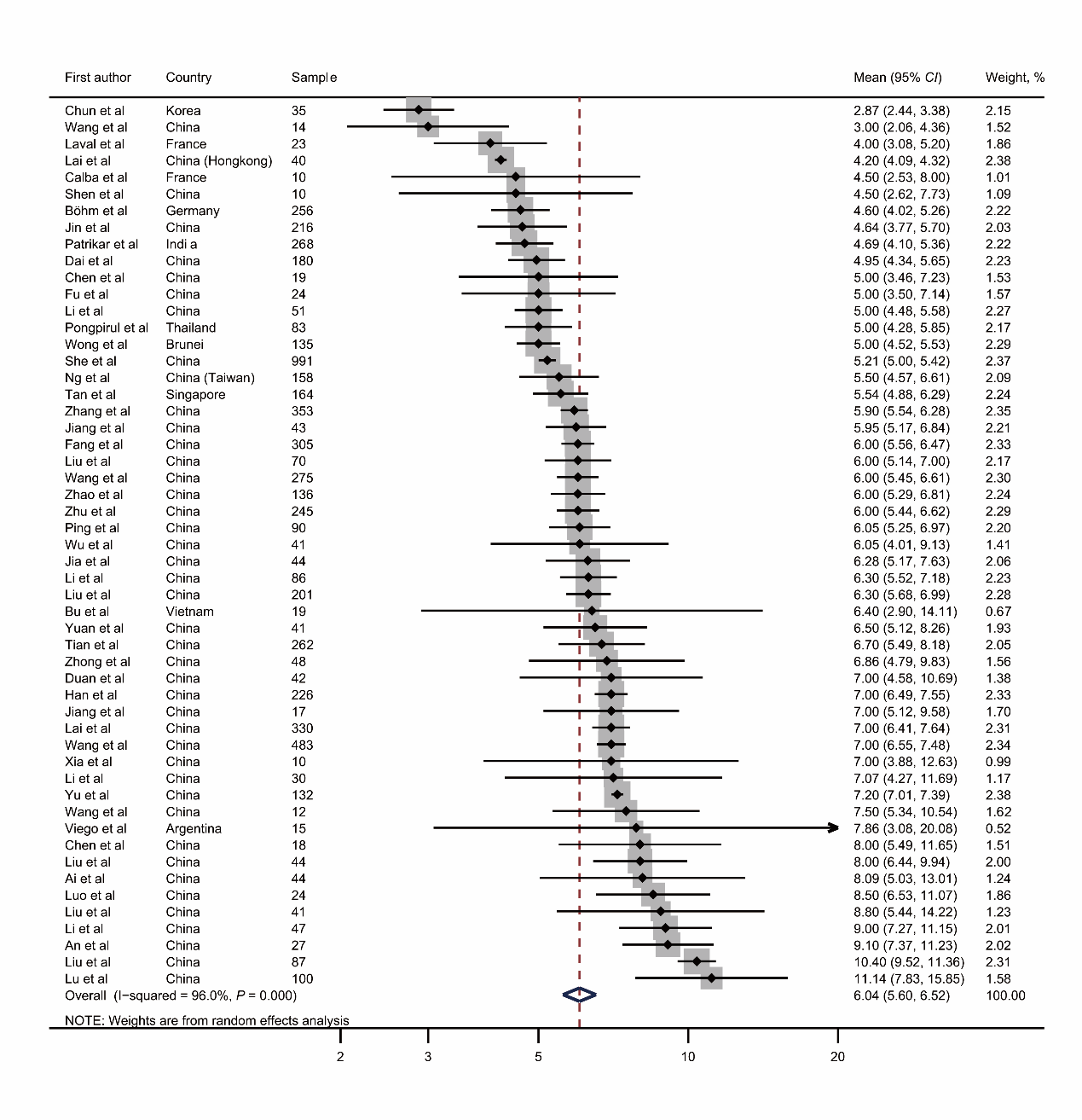


**Figure S1.** The Forest plot for mean incubation period of coronavirus disease 2019 in or outside mainland China. CI, confidence interval.


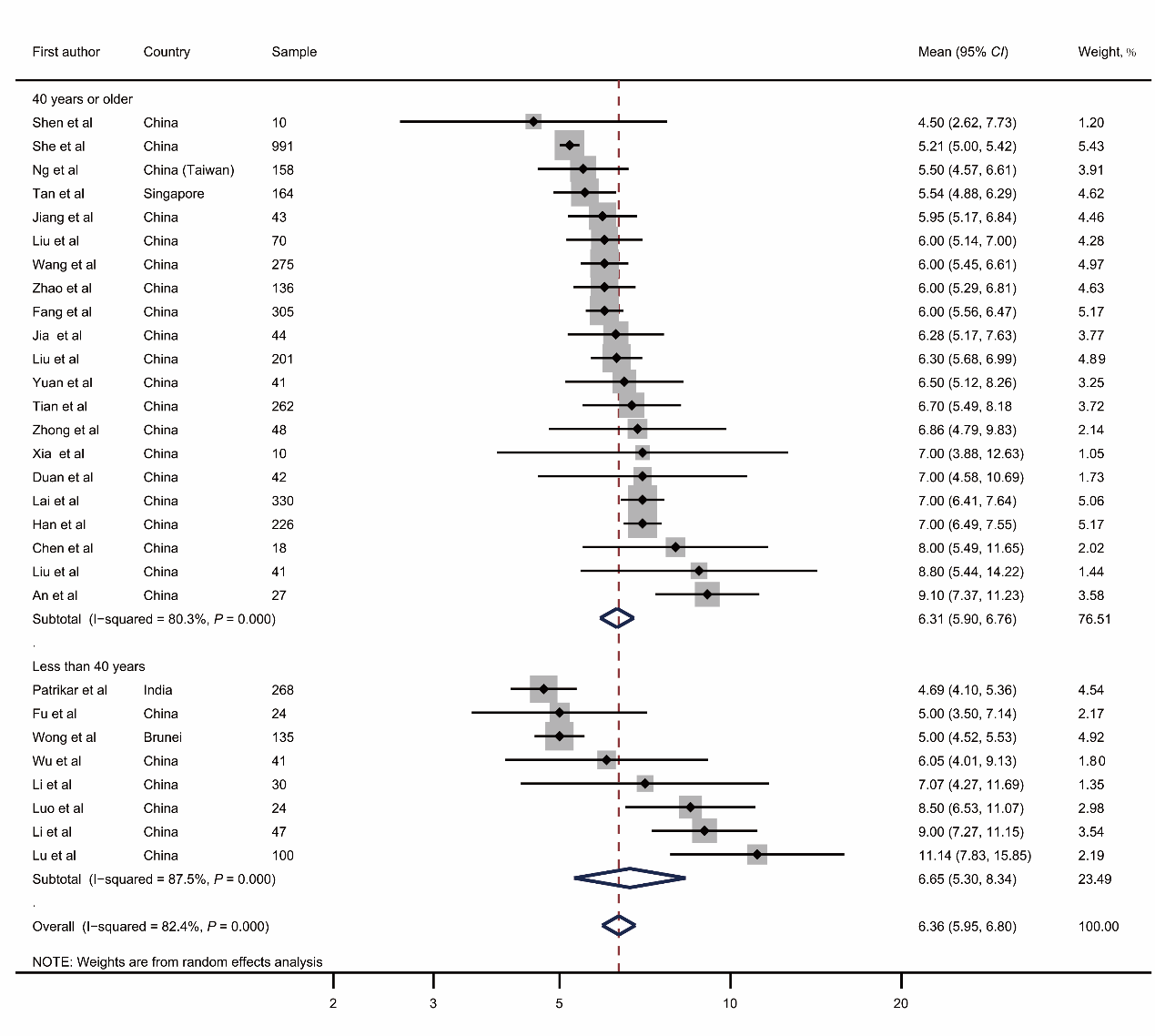


**Figure S2**. The forest plot for mean incubation period of coronavirus disease 2019 by mean age of patients (< 40 years vs. ≥ 40 years). CI, confidence interval.


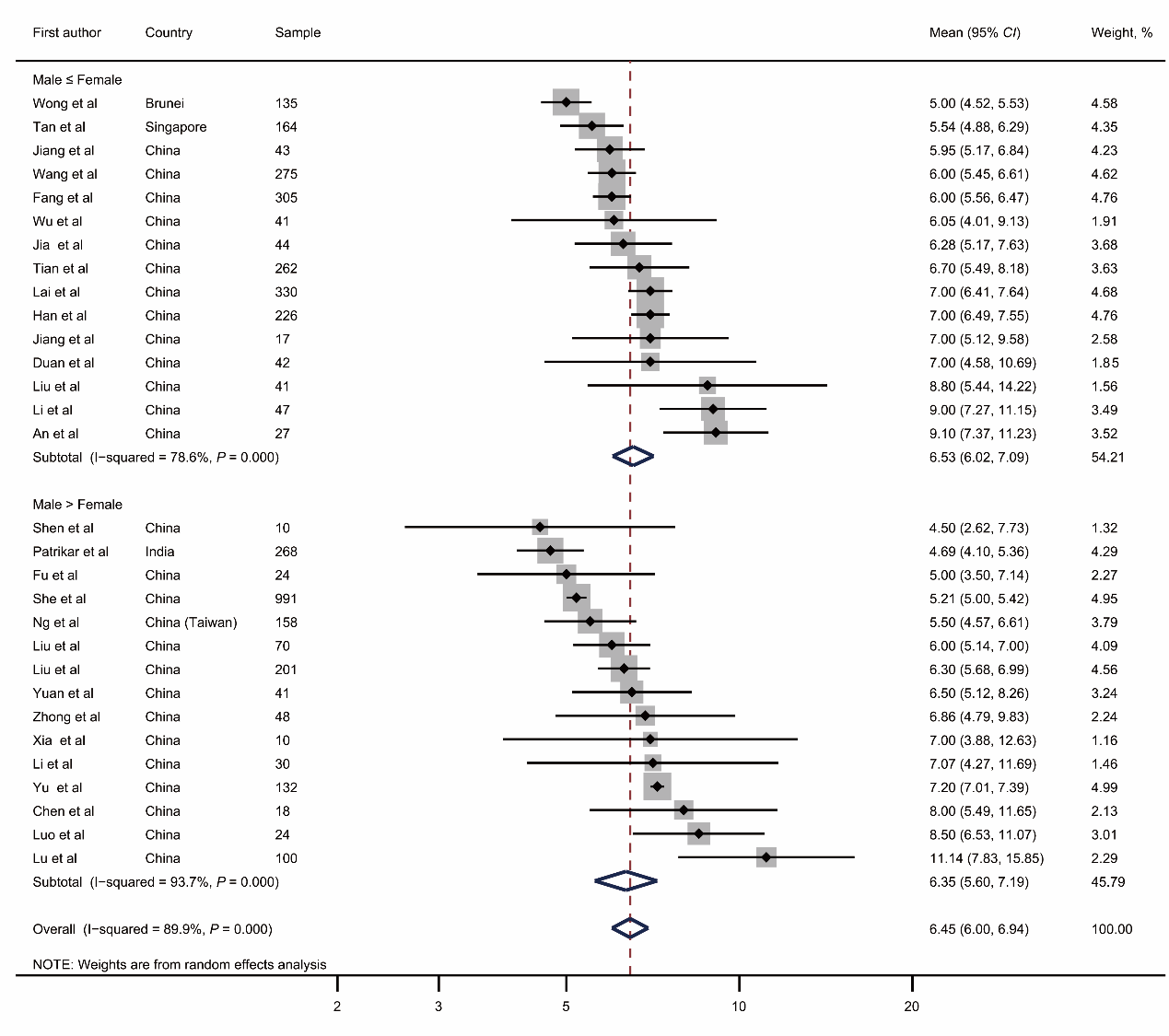


**Figure S3**. The Forest plot for mean incubation period of coronavirus disease 2019 by the ratio of male to female (≤ 1 vs. > 1). CI, confidence interval.


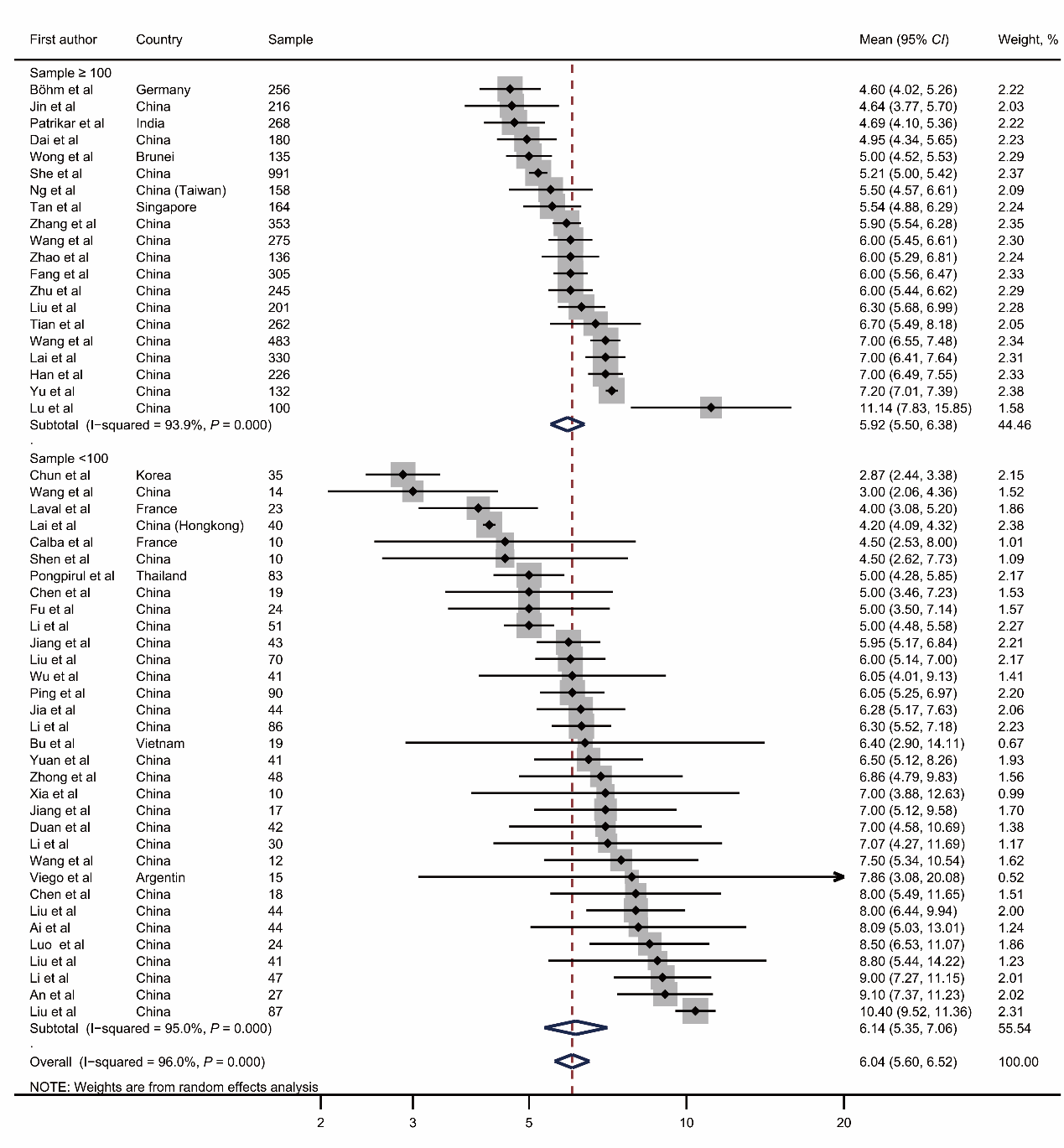


**Figure S4**. The Forest plot for mean incubation period of coronavirus disease 2019 by sample of study (< 100 vs. ≥100). CI, confidence interval.


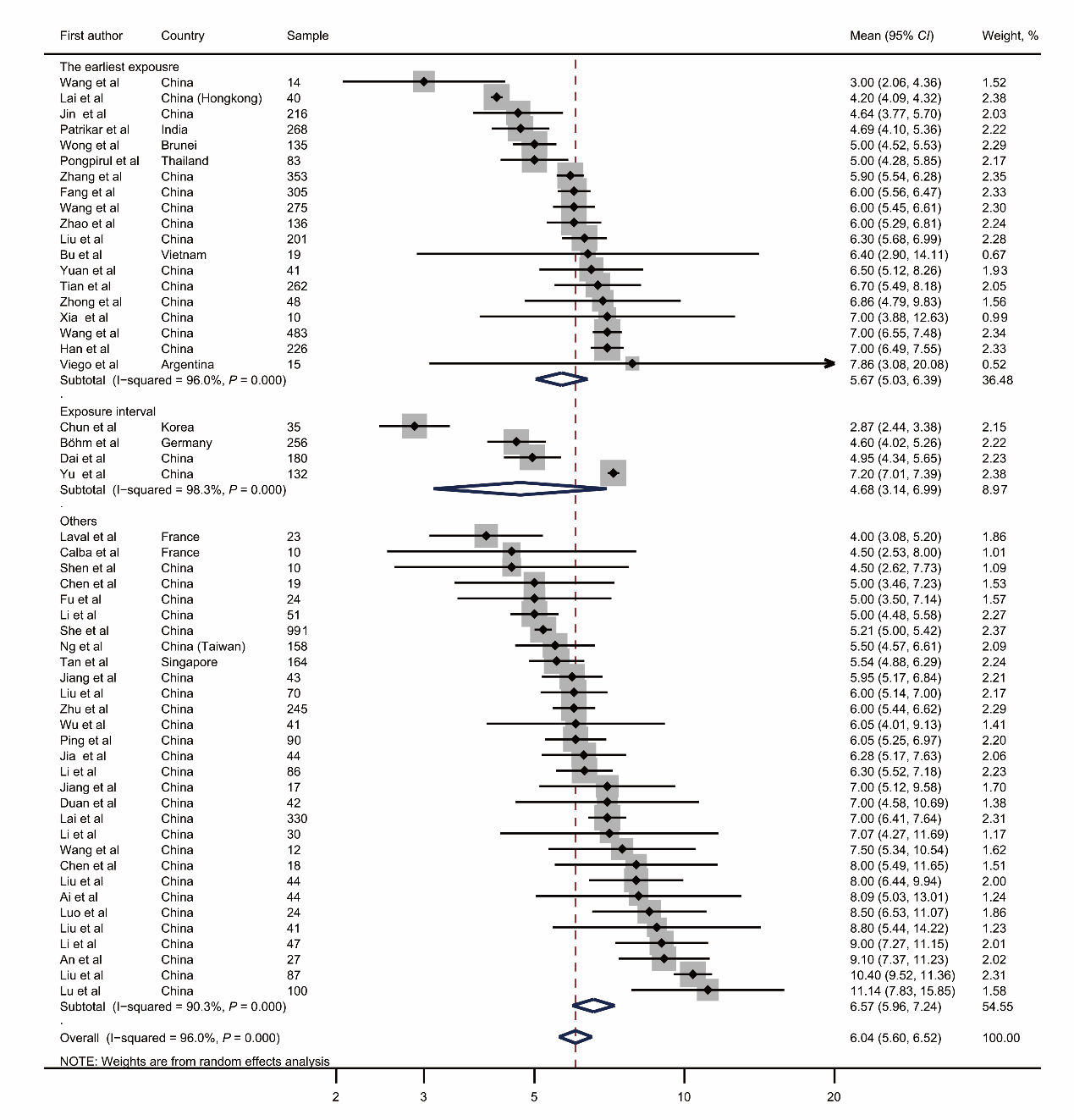
**Figure S5**. The Forest plot for mean incubation period of coronavirus disease 2019 by estimation method of infection date. CI, confidence interval.


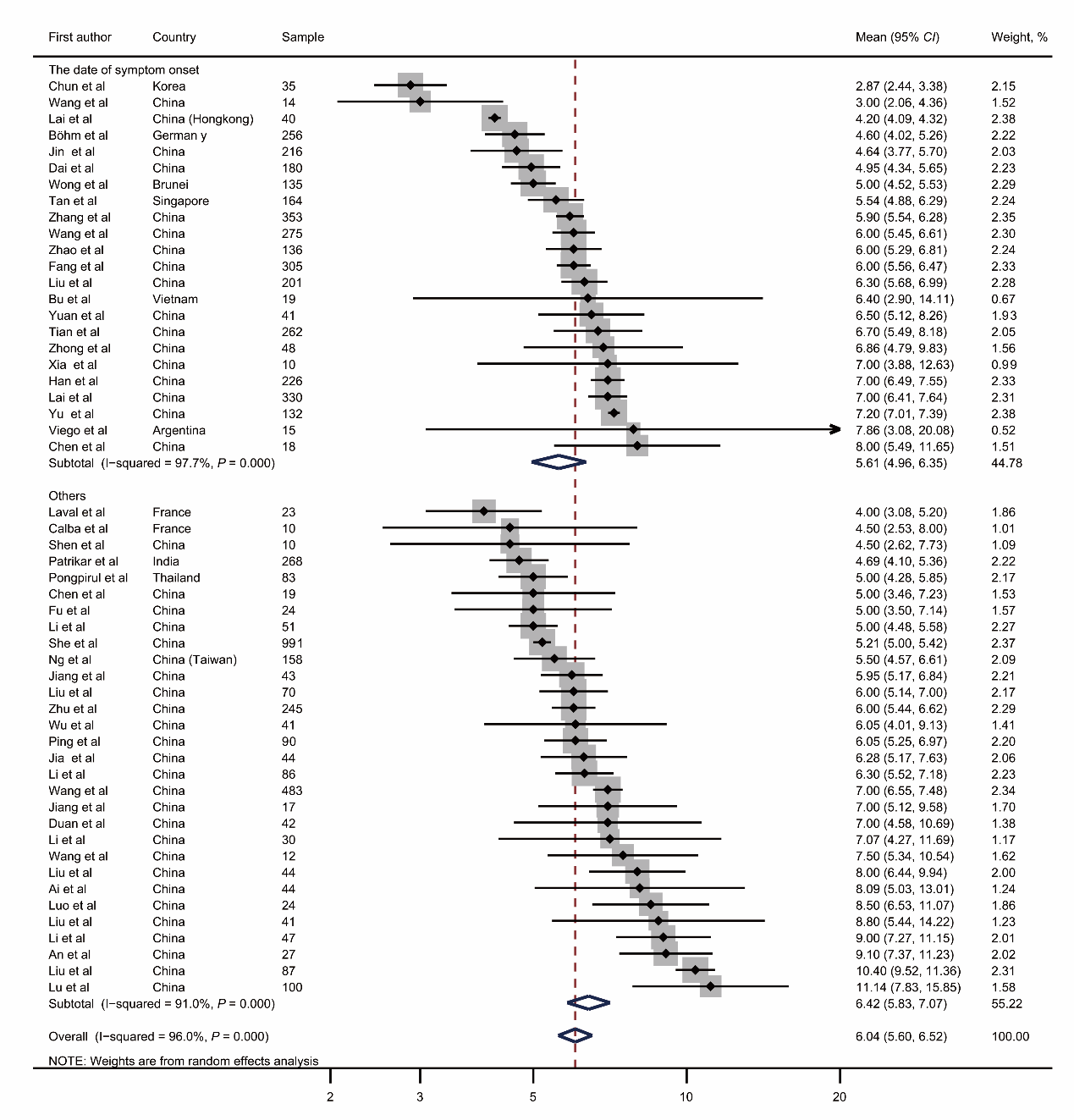
**Figure S6**. The Forest plot for mean incubation period of coronavirus disease 2019 by definition of onset. CI, confidence interval.


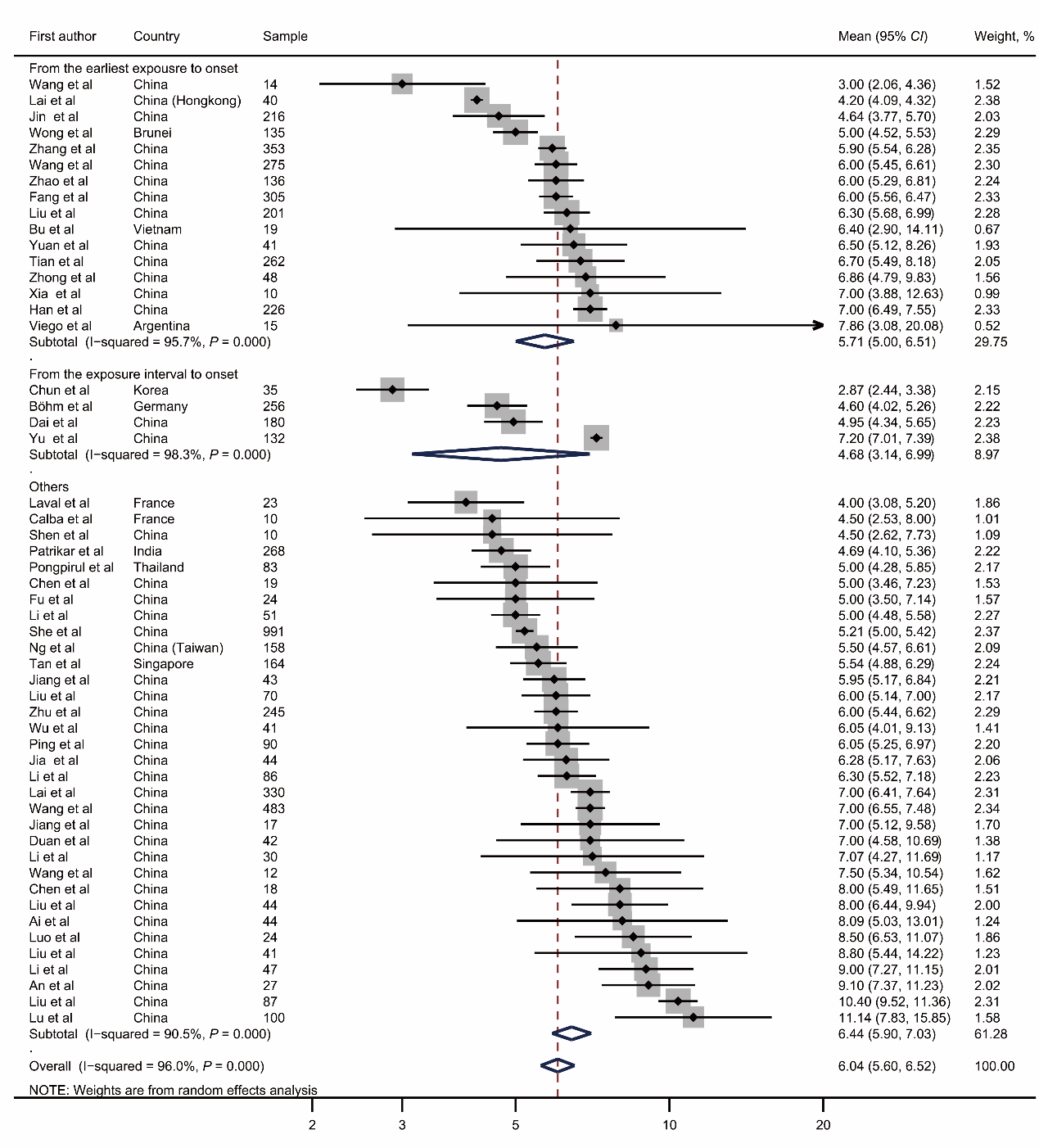
**Figure S7**. The Forest plot for mean incubation period of coronavirus disease 2019 by definition of incubation period. CI, confidence interval.


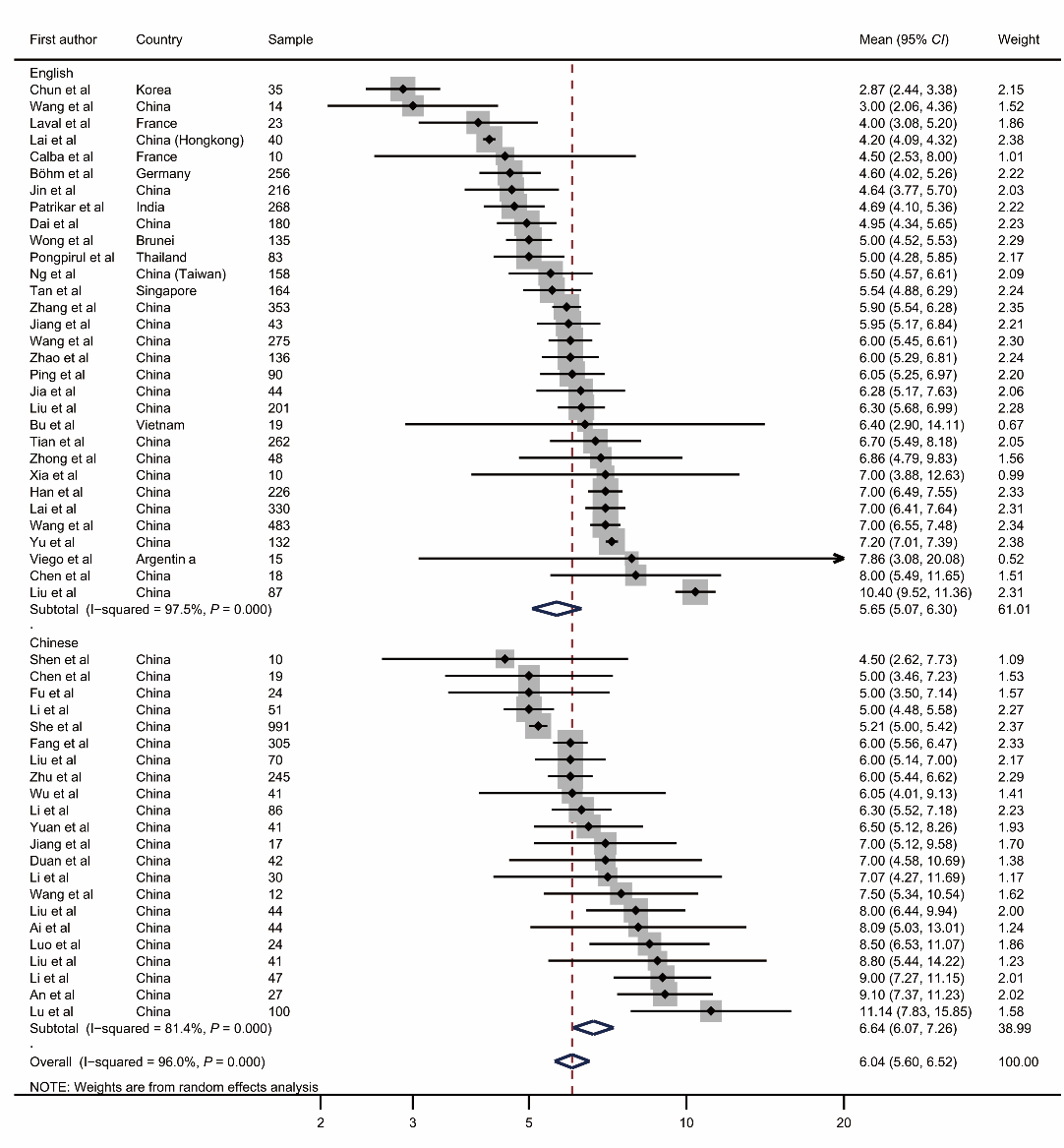


**Figure S8**. The Forest plot for mean incubation period of Coronavirus disease 2019 by publish language. CI, confidence interval.
