# Supplementary figures and images for: The incubation period of COVID-19: a global meta-analysis of 53 studies and a Chinese observation study of 11 545 patients
Source: Infect Dis Poverty. 2021 Sep 17;10:119. doi: 10.1186/s40249-021-00901-9 (PMC8446477; doi:10.1186/s40249-021-00901-9)

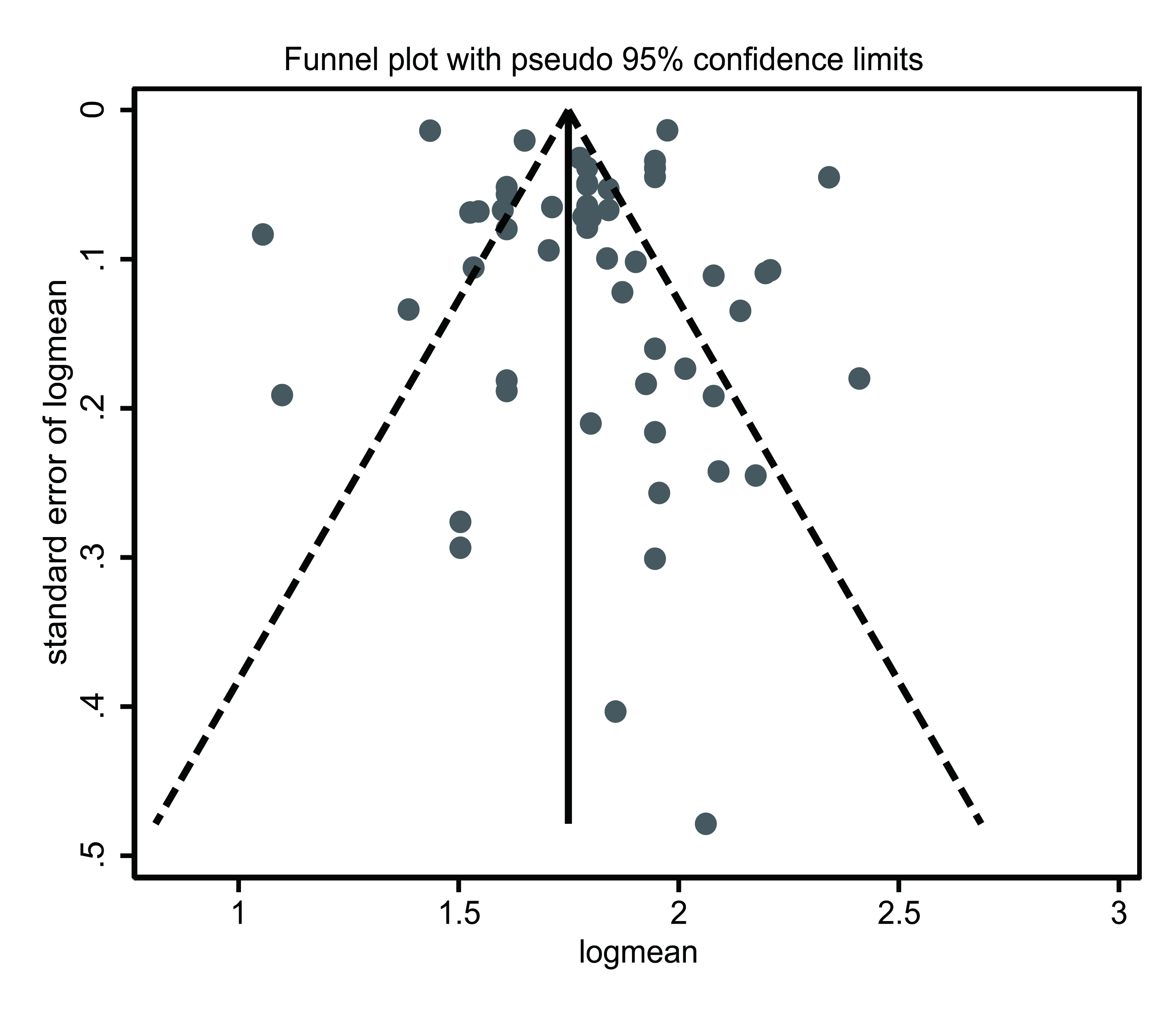

Supplement: Supplementary file 3 — Additional file 3. Additional Figure S9. [file 40249_2021_901_MOESM3_ESM.tif]
