# Supplementary material for: The incubation period of COVID-19: a global meta-analysis of 53 studies and a Chinese observation study of 11 545 patients
Source: Infect Dis Poverty. 2021 Sep 17;10:119. doi: 10.1186/s40249-021-00901-9 (PMC8446477; doi:10.1186/s40249-021-00901-9)
Supplement: Supplementary file 4 — Additional file 4. Additional Figure S10. [file 40249_2021_901_MOESM4_ESM.docx]

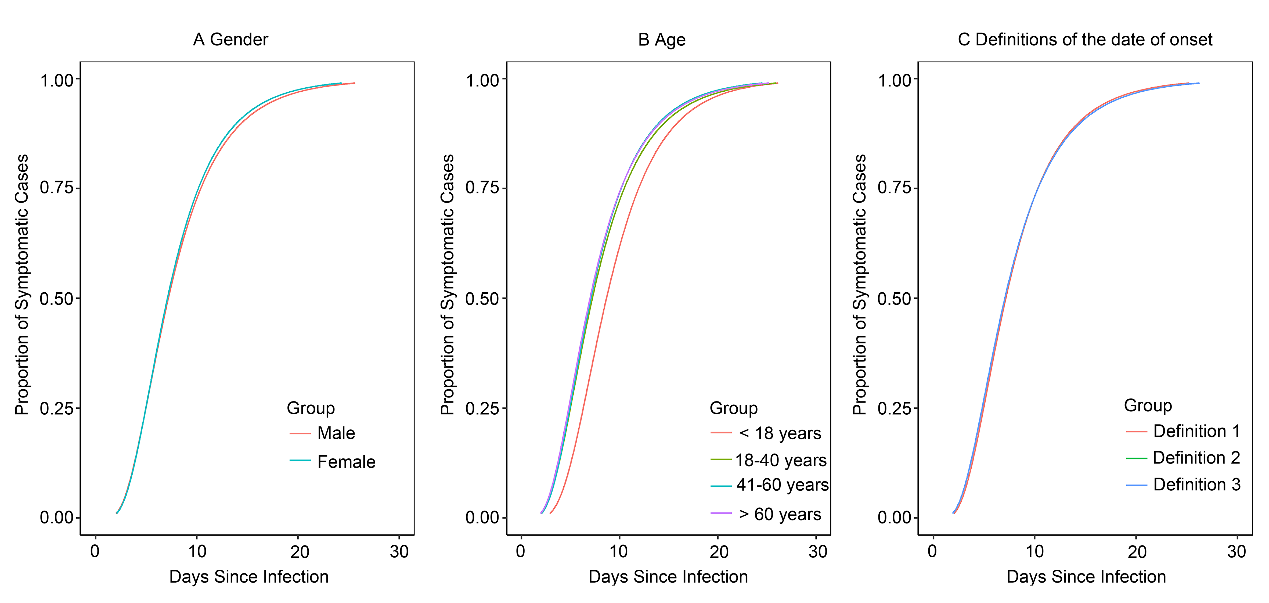


**Figure S10**. The population-specific fitted distribution curve of incubation period of Coronavirus disease 2019 in 11545 patients. A. by gender; B. by age; C. by definition of the date of onset.
